# Supplementary material for: Plasminogen Activator Inhibitor-1 in poorly controlled vs well controlled Type-2 Diabetes Mellitus patients: A case-control study in a district hospital in Ghana
Source: PLoS One. 2021 Apr 15;16(4):e0250090. doi: 10.1371/journal.pone.0250090 (PMC8049243; doi:10.1371/journal.pone.0250090)
Supplement: S1 Questionnaire — (DOCX) [file pone.0250090.s001.docx]

**Plasminogen Activator Inhibitor-1 in poorly controlled vs well controlled Type-2 Diabetes Mellitus patients; a case-control study in a district hospital in Ghana**

**Questionnaire for the Study**

**Introduction**

The purpose of this study is to assess the antigen and activity levels of Plasminogen Activator Inhibitor-1 (PAI-1) among T2DM Ghanaians. You are kindly entreated to respond to all the questions as objectively as you can by ticking the appropriate box. You are assured of strict anonymity and that no content of this information will be disclosed to anyone with reference being made to you.

SUBJECT’S ID: _________ AGE _________________

GENDER _________________ HEIGHT (m^2^) _________

WEIGHT (kg) _________ BMI (kg/m^2^) _____________

SYSTOLIC BP (mm/Hg) _____________ DIASTOLIC BP (mm/Hg) ________________

**Level of education**

Have you ever attended school or any early childhood education programme?

- NO
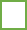
 1. Yes
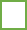


What is the highest degree or level of school you have completed? If currently enrolled, highest degree received?

0. Primary
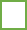


1. Secondary
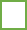


2. Tertiary
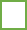


What is your occupation, or what kind of work did you do? __________________

**Alcohol consumption assessment**

Do you consume alcoholic drinks? YES
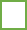
 NO
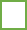


What kind of alcoholic drinks do you consume? (tick those applicable)

Beer
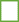
 2. Wine
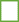
 3. strong liquor
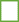


**12. Smoking habits**

Do you smoke?

1) No, I have never smoked
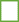


2) No, I have smoked but have given it up
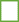


3) Yes, I smoke.
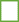


13. When were you first diagnosed with diabetes? Year _________Age_____

14. Any complications of diabetes? (retrieve from records if necessary)

_____________________________________________________________________

**16. Clinical Estimate**

Fasting Plasma Glucose ___________________________

AST ___________________________

ALT ___________________________

Bf for malaria parasite ___________________________

HbA1c ___________________________

Complete Blood Count ___________________________

Lipid profile ____________________________

PAI-1 antigen level (ELISA) ___________________________

PAI-1 activity level (ELISA) ___________________________

*Thank you for your participation*
