# Supplementary material for: Plasminogen Activator Inhibitor-1 in poorly controlled vs well controlled Type-2 Diabetes Mellitus patients: A case-control study in a district hospital in Ghana
Source: PLoS One. 2021 Apr 15;16(4):e0250090. doi: 10.1371/journal.pone.0250090 (PMC8049243; doi:10.1371/journal.pone.0250090)
Supplement: Attachment — Submitted filename: PONE-20-23161_reviewer.docx [file pone.0250090.s003.docx]

The authors provide an interesting and potential important manuscript describing "Plasminogen activator inhibitor-1 in poorly controlled vs well controlled Type-2 diabetes melitus patients; a cross-sectional study in a district hospital in Ghana", The main issues concerning this paper are those concerning the potential associations between PAI-1 and glycaemic.

There are some weak points that need to be addressed by the authors

Major

1. The ratio of male to female in the subgroup is unbalanced, and additional data are needed to explain the gender difference of PAI-1.( see Table1 below)

2. In the table below, abnormal distribution of blood lipids and blood glucose occurred simultaneously between groups. How to determine the relationship between PAI-1 and blood glucose?( see Table2 below)
