# Supplementary material for: Plasminogen Activator Inhibitor-1 in poorly controlled vs well controlled Type-2 Diabetes Mellitus patients: A case-control study in a district hospital in Ghana
Source: PLoS One. 2021 Apr 15;16(4):e0250090. doi: 10.1371/journal.pone.0250090 (PMC8049243; doi:10.1371/journal.pone.0250090)
Supplement: Attachment — Submitted filename: PAI-1 RESPONSE TO EDITOR.docx [file pone.0250090.s004.docx]

Dear Editor,

Thank you for your email. I am pleased to resubmit manuscript number: **PONE-D-20-23161** titled “**Plasminogen Activator Inhibitor-1 in poorly controlled vs well controlled Type-2 Diabetes Mellitus patients; a case-control study in a district hospital in Ghana**" for your consideration.

The concerns raised by the editorial team have been addressed and highlighted below and in the respective sections in the manuscript. I look forward to your favourable response.

Thank you.

1. Please ensure that your manuscript meets PLOS ONE's style requirements, including those for file naming. The PLOS ONE style templates can be found at

<https://journals.plos.org/plosone/s/file?id=wjVg/PLOSOne_formatting_sample_main_body.pdf>  and

<https://journals.plos.org/plosone/s/file?id=ba62/PLOSOne_formatting_sample_title_authors_affiliations.pdf>

 The manuscript meets PLOS ONE’s style requirements.

2. Please include additional information regarding the survey or questionnaire used in the study and ensure that you have provided sufficient details that others could replicate the analyses. For instance, if you developed a questionnaire as part of this study and it is not under a copyright more restrictive than CC-BY, please include a copy, in both the original language and English, as Supporting Information.

 The Questionnaire for the study has been uploaded as a supporting document

3. We noted that you refer to this study as a cross-sectional study throughout the manuscript, but according to your description we would not consider this a cross-sectional study but rather a case control study. In order to avoid confusion we would suggest that you change the wording in your manuscript and avoid referring to this study as a cross-sectional study.

 The ‘cross-sectional study’ wording in the entire document has been replaced with ‘case-control study’

4. Please provide a sample size and power calculation in the Methods, or discuss the reasons for not performing one before study initiation.

 The sample size and power calculation has been included in the Methods

5. Please include captions for your Supporting Information files at the end of your manuscript, and update any in-text citations to match accordingly. Please see our Supporting Information guidelines for more information: http://journals.plos.org/plosone/s/supporting-information.

 Captions have been included in the Supporting Information files

5. Review Comments to the Author

Please use the space provided to explain your answers to the questions above. You may also include additional comments for the author, including concerns about dual publication, research ethics, or publication ethics. (Please upload your review as an attachment if it exceeds 20,000 characters)

Reviewer #1: I found the research to be rigorous, thorough, reasonable, and well-written. I found the investigation as needed for the patients studied and invites’ other researchers to do the same for their patrons. The research agrees with and builds upon prior research.
The wording and spelling conform with U.S. English minus the few words below. These words are listed for the researchers to confirm the spelling is correct for their intended audience.

U.S. English Your Document
etiology aetiology
Analyzed Analysed
Dyslipidemia Dyslipidaemia
Glycemic Glycaemic
Glycosylated Glycosylatedcated
Hematological Haematological
Hemoglobin Haemoglobin
hemostasis haemostasis
Hyperinsulinemia Hyperinsulinaemia
ischemic ischaemic
neighboring	neighbouring

Changes have been made and all of the above words now conform to U.S. English in the manuscript

Reviewer #2: The authors provide an interesting and potential important manuscript describing "Plasminogen activator inhibitor-1 in poorly controlled vs well controlled Type-2 diabetes melitus patients; a cross-sectional study in a district hospital in Ghana", The main issues concerning this paper are those concerning the potential associations between PAI-1 and glycaemic.

There are some weak points that need to be addressed by the authors

Major

1. The ratio of male to female in the subgroup is unbalanced, and additional data are needed to explain the gender difference of PAI-1 (see Table1 below).

This has been rectified and the gender ratio is balanced now

2. In the table below, abnormal distribution of blood lipids and blood glucose occurred simultaneously between groups. How to determine the relationship between PAI-1 and blood glucose? (see Table2 below).

The relationship between PAI-1 and fasting blood glucose has been established and data embedded in the manuscript.
