# Supplementary material for: Plasminogen Activator Inhibitor-1 in poorly controlled vs well controlled Type-2 Diabetes Mellitus patients: A case-control study in a district hospital in Ghana
Source: PLoS One. 2021 Apr 15;16(4):e0250090. doi: 10.1371/journal.pone.0250090 (PMC8049243; doi:10.1371/journal.pone.0250090)
Supplement: Attachment — Submitted filename: Response to PONE-D-20-23161_R1.doc [file pone.0250090.s005.doc]

Dear author

Thank you very much for adjusting the gender ratio. However, the number of men and women remains the same as before, and other data in table1 have not changed. I'm sorry that I cannot accept the explanation for the previous problem. The author should review the data, re-count and collate it.
