# Supplementary material for: Plasminogen Activator Inhibitor-1 in poorly controlled vs well controlled Type-2 Diabetes Mellitus patients: A case-control study in a district hospital in Ghana
Source: PLoS One. 2021 Apr 15;16(4):e0250090. doi: 10.1371/journal.pone.0250090 (PMC8049243; doi:10.1371/journal.pone.0250090)
Supplement: Attachment — Submitted filename: PAI-1 RESPONSE TO EDITOR.docx [file pone.0250090.s006.docx]

Dear Editor,

Thank you for your email. I am pleased to resubmit manuscript number: **PONE-D-20-23161** titled “**Plasminogen Activator Inhibitor-1 in poorly controlled vs well controlled Type-2 Diabetes Mellitus patients; a case-control study in a district hospital in Ghana**" for your consideration.

The concern raised by the editorial team have been addressed and highlighted below and in the respective sections in the manuscript. I look forward to your favourable response.

Thank you.

**Reviewer’s comment**

Thank you very much for adjusting the gender ratio. However, the number of men and women remains the same as before, and other data in table 1 have not changed. I'm sorry that I cannot accept the explanation for the previous problem. The author should review the data, re-count and collate it.

**Authors’ response**

First, the data has been reviewed, recounted and re collated to address the appropriate percentage distribution of the participants’ gender. However, the ratio of male to female in the subgroup still remains unbalanced. It is not possible to balance the gender now, this could have been done at the data collection stage; it cannot be undertaken after data collection. In the methodology we did not indicate anyway where that we controlled for gender (i.e. Collecting equal number of males and females in each arm/sub-group). We therefore have acknowledged this as a limitation of the study.

Second, additional data for comparison of mean PAI-1 activity and PAI-1 Ag level across Gender was analysed and reported as below.

Comparison of PAI-1 activity and PAI-1 Ag level across Gender

The mean PAI-1 activity between males and females were not significantly different (5.25±3.27 vs 5.25±3.90, *p*=0.998). Similarly, no significant differences were observed between PAI-1 Ag level in males and females (25.83±16.85 vs 26.42±21.92, *p*=0.888). With respect to the Type II diabetic group, no significant difference was observed for both PAI-1 activity (5.80±3.90 vs 5.80±4.65, *p*=0.996) and PAI-1 Ag level (28.52±20.13 vs 29.91± 26.15, *p*=0. 822) between males and females. Also, in the control group, PAI-1 activity (4.17±0.63 vs 4.15±0.97, *p*=0.951) and PAI-1 Ag level (20.44±3.38 vs 19.45 ± 3.36, *p*=0.459) were not significantly different across the different groups of gender.

**Table 3: Comparison of PAI-1 activity and PAI-1 Ag across Gender**

|  | **Gender** | | ***p*-value** |
| --- | --- | --- | --- |
| **Both groups** | **Male** | **Female** |  |
| **PAI-1 Activity level (U/mL)** | **5.25 ± 3.27** | **5.25 ± 3.90** | **0.998** |
| **PAI-1 Ag level (ng/mL)** | **25.83 ± 16.85** | **26.42 ± 21.92** | **0.888** |
| **T2DM Participants** |  |  |  |
| **PAI-1 Activity level (U/mL)** | **5.80 ± 3.90** | **5.80 ± 4.65** | **0.996** |
| **PAI-1 Ag level (ng/mL)** | **28.52 ± 20.13** | **29.91 ± 26.15** | **0.822** |
| **Healthy Controls** |  |  |  |
| **PAI-1 Activity level (U/mL)** | **4.17 ± 0.63** | **4.15 ± 0.97** | **0.951** |
| **PAI-1 Ag level (ng/mL)** | **20.44 ± 3.38** | **19.45 ± 3.36** | **0.459** |

**Data are presented as Mean ± standard deviation (SD); PAI-1= Plasminogen activator inhibitor-1; Ag= Antigen**

Acknowledged limitations of the study.

The study was limited by our inability to determine the specific polymorphisms of the PAI-1 gene in the T2DM subjects. Also, the ratio of male to female in both subgroups were unbalanced, the non-significant difference may have been masked by the unbalanced gender. To help address this limitation we, recommend that participants be matched for age and gender in future studies.
